# Pneumococcus and the Elderly in Italy: A Summary of Available Evidence Regarding Carriage, Clinical Burden of Lower Respiratory Tract Infections and On-Field Effectiveness of PCV13 Vaccination

**DOI:** 10.3390/ijms17071140

**Published:** 2016-07-15

**Authors:** Andrea Orsi, Filippo Ansaldi, Cecilia Trucchi, Roberto Rosselli, Giancarlo Icardi

**Affiliations:** 1 Department of Health Sciences (DiSSal), University of Genoa, 16132 Genoa, Italy; filippo.ansaldi@unige.it (F.A.); cecilia.trucchi@edu.unige.it (C.T.); icardi@unige.it (G.I.); 2 IRCCS AOU San Martino-IST, 16132 Genoa, Italy; 3 ASL3 Genovese, 16132 Genoa, Italy; roberto.rosselli@asl3.liguria.it

**Keywords:** Streptococcus pneumoniae, pneumococcal conjugate vaccines, PCV13, elderly, pharyngeal carriage, CAP, LRTI, effectiveness

## Abstract

Streptococcus pneumoniae is currently the leading cause of community-acquired pneumonia (CAP) and lower respiratory tract infections (LRTI) in adults, elderly and high-risk subjects worldwide. The clear benefits of pneumococcal conjugate vaccination in childhood have been accompanied by a decrease of vaccine-serotype invasive diseases among adults in several countries, mainly due to the herd effect mediated by the reduction of vaccine-serotype nasopharyngeal colonization in both age groups, but this reduction in the incidence of pneumonia has not been observed. The “Community Acquired Pneumonia Immunization Trial in Adults” (CAPITA) study provided conclusive evidence about 13-valent pneumococcal conjugate vaccine (PCV13) efficacy in preventing CAP in adults and led Western countries to issue new recommendations for pneumococcal immunization targeting subjects >50 years and high-risk groups, with marked differences with respect to age and/or risk groups immunized, eligibility for reimbursement and national, regional or local implementation. Several Italian regions implemented PCV13 immunization programs in adults and interesting data have been come available in the last years, especially from Liguria, a Northern region with a high and long-lasting pneumococcal vaccine immunological pressure in infants. In this review, currently available evidence from Italy and Liguria regarding pneumococcal carriage, burden of CAP and LRTI, and on-field effectiveness of PCV13 immunization in adults and elderly will be summarized.

## 1. Introduction

Streptococcus pneumoniae (Sp) is currently the leading cause of community-acquired pneumonia (CAP) and lower respiratory tract infections (LRTI), two important clinical pictures with a heavy burden in terms of morbidity and mortality especially among adults, elderly and high-risk subjects worldwide, accounting for 25% to 50% of the cases depending on the diagnostic assay, geographic area, and setting [1,2,3,4,5,6,7,8,9,10]. Thanks to the extensive use of pneumococcal multi-valent conjugate vaccines in the pediatric population, starting from 2000 in the US, Canada and several South-American and European countries including Italy, a sensational decline in invasive (IPD) and non-invasive Sp-related diseases sustained by vaccine serotypes in children has been observed [11,12,13,14,15,16]. Multiple advantages have derived from the specific technology of pneumococcal conjugate vaccines, compared to first-generation vaccines based on polysaccharides used as antigens [17]. Conjugate vaccines are immunogenic in young children, the elderly and immune-compromised hosts, and induce immunologic memory and boostability. Higher antibody avidity and avidity maturation are elicited by conjugate vaccines, with a more effective action in mediating protective functions. Moreover, conjugate vaccinations can induce herd immunity by reducing the nasopharyngeal carriage of Sp through different mechanisms mainly involving the induction of IgA mucosal antibodies, high-titered serum antibodies transudating into naso- and oropharynx and Sp specific anticapsular IgG serum concentrations [18,19,20,21,22,23,24]. The clear benefits of the widespread use of pneumococcal conjugate vaccination in childhood have been accompanied by a decrease in the rates of vaccine-serotype IPD among adults in several countries, mainly due to the herd immunity effect mediated by the reduction of vaccine-serotype nasopharyngeal colonization in both age groups [11,25,26]. This reduction in the incidence of pneumonia among adults has not been observed: Nelson et al.; by conducting a population-based pneumonia surveillance in USA before and after infant vaccine introduction in 2000, observed a decline in rates of both outpatient and hospitalized pneumonia in children less than 1 year of age following vaccine introduction, but no consistent reductions in pneumonia rates among older children and adults were recorded [27].

So far, the Sp related disease burden, particularly due to CAP and LRTI, has remained very high, especially in the elderly and high-risk groups, and in the last few years this evidence has lead several countries to issue recommendations for pneumococcal vaccination in adults, outside routine immunization programs in infants and children, already recommended by the World Health Organization (WHO) since 2006 [28,29]. Two types of vaccines are currently available for the prevention of pneumococcal diseases in adults: the 23-valent pneumococcal polysaccharide vaccine (PPV23), and the 13-valent pneumococcal conjugate vaccine (PCV13). Recommendations for pneumococcal immunization across the world, and in particular in Europe, are complex, heterogeneous and frequently updated, differing with regards to age groups and/or risk groups immunized, different types of available vaccine advised for different groups, eligibility for reimbursement and national, regional or local implementation [28]. This heterogeneity has a heavy impact on vaccination coverage rates, and marked differences are observed among various countries [30,31,32].

Since 2005, the Italian National Vaccination Plan has recommended the administration of PPV23 to subjects of any age that present high-risk conditions or comorbidities. The last available edition of the Plan (2012–2014) reported that PCV13 was not indicated for adult vaccination at the moment and that the extension of the indications to adult age was expected [33]. Nevertheless, following the PCV13 approval for use among adults aged ≥50 years to prevent pneumonia and invasive disease caused by Sp serotypes contained in the vaccine, several Italian regions implemented age-based pneumococcal immunization programs in adults and the elderly, recommending a single dose of PCV13 or a dose of PCV13 first, followed by a dose of PPSV23 [28]. In Liguria—a Region of nearly 1,700,000 inhabitants in North-Western Italy—a universal childhood vaccination programme against Sp was started in May 2003, and pneumococcal conjugate vaccines uptake (7-valent until autumn 2010 and then 13-valent) reached a coverage of >80% and >90% in all districts since 2004 and 2007, respectively, determining an epidemiological picture consisting of a very high and long-lasting vaccine immunological pressure, which is unusual for Europe [34].

With regard to pneumococcal vaccination in adults, in 2000 Liguria launched a large-scale programme of vaccination with PPV23 in all individuals over 64 years of age and in high risk groups, in particular those with an increased likelihood of acquiring pneumococcal infection and those presenting with a serious risk of complications (asthma, chronic obstructive pulmonary disease (COPD), bronchitis, chronic respiratory disease, cardiovascular disease, chronic renal failure, diabetes mellitus, immunodeficiency and functional or anatomic asplenia). Although no data are available about PPV23 uptake for the Liguria Region, cumulative coverage rates calculated in a region bordering Liguria in 2007, ranged from 26% to 31% in the elderly and was approximately 23% in adults with underlying risk conditions [35]. Since 2013, Liguria has implemented a new recommendation for pneumococcal immunization in adults, offering PCV13 and PPV23 in series in subjects aged between 70 and 75 years, and in individuals with specific co-morbidities that are considered to place them at an increased risk of pneumococcal diseases, regardless of age [36].

In the following sections, currently available evidence from Italy and Liguria regarding pneumococcal carriage, clinical burden of pneumococcal-related diseases and on-field effectiveness of PCV13 immunization programs in adults and the elderly will be summarized.

## 2. Mechanisms of Protection Afforded by Pneumococcal Conjugate Vaccines

Both pneumococcal plain polysaccharide and conjugate vaccines rely on the capsular polysaccharide antigen to induce a serotype-specific immune response. The plain polysaccharides are T-cell-independent antigens that do not allow an expansion of serotype-specific B cells, or the creation of serotype-specific memory B cells, whereas conjugated vaccines elicit a T-cell-dependent immune response, due to the processing of the carrier protein within specific naive B cells, together with the polysaccharide antigens [37,38]. Via MHC class II molecules, the peptides are presented to carrier-peptide-specific helper T cells, which enhance the immune response provided by the B cells and induce specific memory B cells, ensuring an antibody response of greater specificity and functionality and a good immunologic memory and boostability.

Another fundamental feature of pneumococcal polysaccharide conjugate vaccines is the ability to decrease the nasopharyngeal carriage of Sp. Although the specific mechanisms of action are not completely understood yet, it is well accepted and widely demonstrated that the use of conjugate vaccines has reduced the pneumococcal carriage rates in the infant population. Pharyngeal colonization is considered a key step for the development of pneumococcal infections [39]. Moreover, pneumococcal carriage is an important determinant of horizontal spread of Sp strains within the community, favoured by crowding, especially in hospitals, day-care centers and prisons. Therefore, one of the pillars of an effective pneumococcal immunization strategy should be the prevention of nasopharyngeal colonization.

## 3. Estimates of Pneumococcal Carriage in Adults and the Elderly

The evaluation of carriage characteristics represents a relevant tool for monitoring the risk of the development of pneumococcal infection and the impact of an immunization programme in both vaccinated and unvaccinated subjects [40,41]. In particular, pneumococcal carriage may enable to monitor how vaccination affects circulating pneumococcal serotypes. Most of the available studies about carriage were conducted in paediatric age groups. So far, the epidemiological picture of serotypes carried in adults has been poorly investigated. Increasing knowledge on this matter is of great interest, in the light of the high and long-lasting immunological pressure due to conjugate vaccine coverage in children, the availability and use of the PPV23 vaccine, and the recent introduction of PCV13 for adult and elderly immunization. Before the introduction of pneumococcal conjugate vaccine into the immunization schedule of infants and children, studies performed in developed countries showed highly variable colonization rates among adults, with values between 1.5% and 30%. However, comparisons are difficult to make due to different laboratory methods, vaccine coverage in the paediatric population, and behavioural issues of enrolled subjects [42,43,44,45,46]. An evaluation performed among adults in the Netherlands, after the introduction of pneumococcal conjugate vaccine in infants, reported a prevalence of carriers that was >30% in adults aged 60–89 years. Interestingly, frequencies of vaccine-serotypes detected among elderly carriers closely resemble the frequencies of corresponding pneumococcal serotypes reported in vaccinated infants in a previous surveillance study [47].

Currently available Italian data for pneumococcal pharyngeal colonization in adults derive from two studies, both performed before implementing the use of PCV13 in this age group [48,49]. Ansaldi et al. [48] conducted a serial cross-sectional study of nasopharyngeal carriage among adults aged 60 years or over in winter-spring 2012 in Genoa, the capital of the Liguria region, with the aim to describe circulating Sp serotypes and compare the adult pattern with the one observed in a previous experience in children, performed in the same area one year before, by using the same sampling and laboratory methods. Among the 283 enrolled adults, the adjusted carriage prevalence of participants with at least one positive sample was 18.7%, with the 70–79 year age group showing a proportion of positive individuals that was more than twice the one observed in older adults. Among serotypes included in PCV13 composition and not included in the 7-valent conjugate vaccine, serotypes 5, 19A, and 3 showed the highest prevalence, while among PPV23 serotypes, 10A and 9N/9L were the most prevalent. Through a multivariate approach, Authors demonstrated that contact with children attending day care doubled the carriage prevalence, and belonging to the 70–79 year group increased carriage prevalence 2.5-fold with respect to subjects aged ≥80 years. A strong monotonic correlation was observed between the prevalence of the 15 most prevalent serotypes recorded in adults aged 60 years or over and that observed in children younger than 5 years, supporting the hypothesis of a possible pneumococcal exchange between grandparents and grandchildren and the crucial role played by the pediatric reservoir.

Esposito et al. [49] screened for pneumococcal colonization status 417 subjects aged ≥65 years who regularly attended two centres for older adults in Milan, capital of the Lombardia region, by adopting different a laboratory procedure for the identification of Sp, compared to Ansaldi et al. Forty-one patients (prevalence = 9.8%) were found to be pneumococcal carriers, and colonization was significantly less common among individuals who had underlying comorbidities compared to those who did not. Interestingly, individuals <75 years old were more common among pneumococcal carriers than among negative subjects, although a statistically significant difference was not reached. Serotypes 19F, 24 and 15 were the most prevalent ones, and carriers of only PCV13 serotypes were less common to show co-morbidities, in particular respiratory allergies and cardiac underlying diseases, when compared with subjects who were positive only for non-PCV13 serotypes.

Both studies underlined that pneumococcal carriage in the elderly is not a rare event and that new PCV13 recommendations could have a valuable impact in the prevention of pneumococcal diseases in older people.

## 4. Clinical Burden of CAP and LRTI in Adults

The elderly are significantly affected by CAP and LRTI worldwide, experiencing high rates of access to primary care services, hospitalization, and mortality [3,4,5,6,7,8]. In developed countries, this scenario is worsened partly due to the overall increase of people aged >65 years and to the prolonged survival of subjects with chronic diseases or other co-morbidities [9,50,51,52]. Sp is considered the leading etiologic agent of adult CAP throughout the world, accounting for 30%–50% of CAP cases requiring hospitalization in adults living in developed countries [8,53,54,55]. However, available evidence on the burden of CAP and LRTI in the elderly are largely incomplete and vary significantly across studies, which does not enable to accurately quantify this clinical picture [56,57,58,59].

Different factors may explain the variation of available CAP and LRTI estimates, in particular differences (i) in the performances of the surveillance system used to detect LRTI cases; (ii) in the definition of pneumonia (radiologically vs. clinically defined, involving outpatients and/or hospitalized patients); and (iii) in the study design [60]. However, considering epidemiological data as the cornerstone of surveillance of infectious diseases, and in the light of the introduction of an effective vaccine for the prevention of pneumococcal pneumonia in adults, population-based evaluations of the real burden of LRTI and pneumonia are needed to support institutional decisions regarding the best immunization strategy, and to monitor the impact of the new vaccination programs. In Italy, Sp infections are not included in the national reporting system as mandatory notifications and a nationwide sensitive surveillance system of pneumococcal diseases, including CAP and LRTI, is currently lacking. So far, only some regional experiences are available, conducted by using very different methodologies, and data regarding the epidemiology of CAP and LRTI in adults are very limited. Viegi et al. [61] reported the first and unique estimate of CAP incidence on the entire national territory, by recording new cases of suspected CAP observed by a random sample of general practitioners living in 40 Italian provinces, in the period between 15 February 1999–14 February 2000. The overall annual CAP incidence per 1000 inhabitants was equal to 1.703, slightly higher in women than in men, in Northern than in Central-Southern Italy, and showing an increase with ageing ranging from 0.734‰ in children less than 14 years up to 3.338‰ in the elderly (64+ years).

Giorgi Rossi et al. evaluated the burden of hospitalized pneumonia in adults in the Lazio region from 1997 to 1999, by analysing hospital discharge records from all the Lazio hospitals. The annual incidence rate for CAP was 1.58 episodes per 1000 inhabitants, ranging between 0.78 (for those aged <65 years) and 4.8 (for those aged ≥65 years). The aetiology was reported for only 20% of CAP episodes and Sp was the most frequent microorganism [62]. Both of these studies were conducted before the implementation of universal childhood immunization programs against Sp. In recent years, Amodio et al. [63] estimated the burden of pneumococcal pneumonia on the hospitalizations of patients aged ≥50 years and residing in Sicily, in the period between 2005 and 2012, using the discharge records of all of the hospitals in Sicily. The authors observed the mean annual incidence rates for all-cause pneumonia and unspecified pneumonia, which were 4.97 and 3.83 cases/1000 inhabitants, respectively, with an increasing trend over time. Instead, model estimated pneumococcal pneumonia entailed a mean hospitalization rate of 1.13/1000 inhabitants, with a statistically significant decreasing temporal trend. Hospitalization rates for pneumococcal pneumonia were correlated with age, where the risk increased about 15-fold from age class 50–54 years to ≥80 years. Bechini et al. investigated acute hospital admission rates for diseases potentially due to Sp, by performing a population-based retrospective study in elderly people aged ≥65 living in Florence, capital of the Toscana region, from 1 January 2010 to 31 December 2012. Among hospitalizations potentially attributable to pneumococcus in the elderly population, pneumonia was the most frequent cause of hospitalization, where the trend clearly increased with the increase of age [64]. Ansaldi et al. [65] estimated the LRTI burden among subjects >18 years resident in the metropolitan area of Genoa, capital of the Liguria region, in terms of emergency department (ED) accesses recorded by a syndrome surveillance system operating at the main hospitals of the city and accounting for nearly 70% of the adult population ED accesses. The surveyed period covered 3 years (from September 2010 to August 2013) and ended when the new regional PCV13 recommendations for adults were implemented. Incidence of hospital access for LRTI was stable during the 3 seasons and in the 18–64 year age group, ranging between 1.5 and 2.3 accesses/1000 inhabitants. The highest incidences were observed among subjects aged 80–84 years and >84 years, with values ranging between 8.3 and 14.7 accesses/1000 population, respectively. Chronic respiratory and cardiovascular diseases, chronic kidney failure, transplantation and immunosuppression represented the most frequent risk factor observed among subjects who had an ED access for LRTI. The incidence of ED accesses in these patients was 1.4–31.9-fold higher in comparison with that observed in subjects without cited comorbidities, according to the age group and the disease.

The reported results highlighted a relevant and strongly age- and risk factor-related burden of LRTI and pneumonia, although a large variability among published studies exists. Major benefits from the availability of an effective vaccine for adults in the prevention of pneumococcal pneumonia will be expected from a “risk-based” strategy coupled by an “age-based” vaccination, in order to give priority to patients with the highest risk of developing pneumococcal-related diseases and complications, and to overcome the well-known difficulties to reach these populations and an adequate vaccine coverage rate.

## 5. Evaluations of On-Field Effectiveness of PCV13 Immunization in Adults

The results of the “Community Acquired Pneumonia Immunization Trial in Adults” (CAPITA) study provided conclusive evidence about the efficacy of PCV13 in preventing vaccine-type CAP in adults. However, estimates of on-field effectiveness of large national or regional immunization programs are needed to identify an appropriately targeted immunization strategy that optimizes the vaccine effect, and support decision-makers with reliable and updated data [66,67]. In particular, population-level impact studies analysing changes, between the pre-vaccination and post-vaccination periods, in the incidence of Sp-related endpoints in adults and elderly people, target of PCV13 immunization, are essential considering the methodological criticisms in the evaluation of pneumococcal conjugate vaccine effectiveness [60]. Observational studies, such as cohort and case-control analysis, are logistically easier and cheaper compared to randomised clinical trials, but the effectiveness of the results could be affected by various biases, and a careful check of potential confounders should be performed.

**Figure 1:**
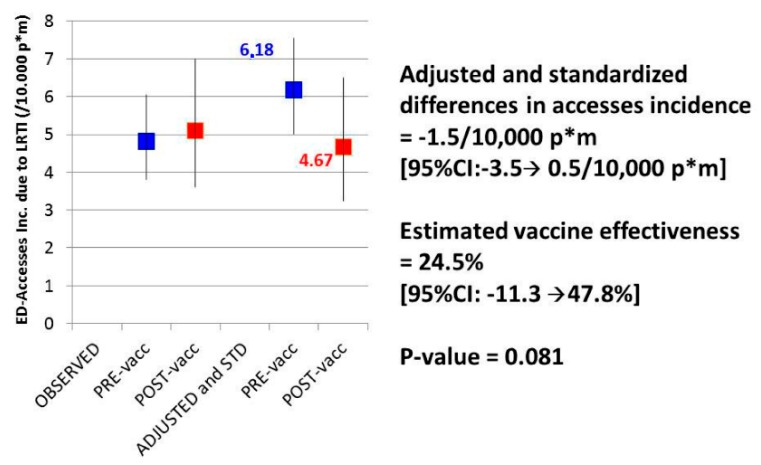
Observed and corrected incidences of Emergency Department (ED) accesses for lower respiratory tract infections (LRTI) before and after 13-valent pneumococcal conjugate vaccine (PCV13) immunization, in a cohort of 70–75 year-old Genoa inhabitants in 2010–2014.

To the best of our knowledge, the only Italian published experience reporting an estimate of on-field effectiveness of a PCV13 immunization program implementation in adults is that of Orsi et al. [36]. Our research group reported the preliminary results of a crossover evaluation of the effect of PCV13 introduction in adults aged ≥70 years in Liguria, conducted in Genoa in the period between September 2010 and August 2014 and is still ongoing. Vaccine effectiveness was estimated in terms of incidence of ED-access for LRTI obtained by the above cited syndrome surveillance system, measured before and after PCV13 vaccination in a population cohort of more than 30,000 individuals aged between 70 and 75 years at the beginning of the study and resident in the metropolitan area of Genoa. Updated preliminary estimates, based on an observation period of 155,274 and 74,419 person-months, respectively before and after pneumococcal vaccination, of the first 3782 vaccinated subjects (median age at the time of vaccination 74.6 years, 10°–90° perc. 70.6–76.3 years, median time of observation before and after vaccination 41.8 and 18.9 months, respectively), showed a reduction in the incidence of ED accesses for LRTI in the vaccinated population, compared to those not vaccinated. The preventive fraction, adjusted for ageing effects and standardized for the different incidences observed during the study period, was estimated to be 24.5% (95% CI: −11.3%–47.8%), with a decrease in ED access incidence of 1.5/10,000 person-months (95% CI: −3.5–0.5/10,000 person-months) (Figure 1). The surveillance period ended on August 2015 and definitive results will be available in the second half of 2016, when the expected vaccinated cohort will add to about 6000 subjects. This population-based approach showed the effectiveness of the current recommendations of the Liguria region for the prevention of pneumococcal disease in adults and high-risk groups, and the beneficial impact of PCV13 vaccination in adults in “real world” clinical and epidemiological settings.

## 6. Future Perspectives

Immunological pressure and herd effect due to the high and long-lasting PCV13 coverage rates among children will probably continue to reduce pneumococcal disease burden in the community and also among adults and the elderly. Therefore, the maximum benefit of PCV13 use in adults is to be expected in the first years post-introduction and it is likely to be reduced substantially in the long-term [68]. For these reasons, the US Advisory Committee on Immunization Practices (ACIP) recently established to re-evaluate and revise the recommendations for PCV13 and PPV23 use in older adults ≥65 years old, if needed, in 2018 [29].

Surveillance of the impact of PCV13 use in target adult populations and in the community remains a fundamental tool in the fight against the disease: vaccine uptake should carefully be monitored, together with changes in the distribution of serotypes responsible for pharyngeal colonization and causing pneumococcal disease among both children and adults. In addition, data from observational studies on on-field effectiveness of PCV13 among adults and elderly people are needed, monitoring both invasive diseases and non-bacteremic pneumococcal pneumonia.

Future research will address a number of critical or unclear issues. For instance, little is known about the duration of protection for conjugate vaccines among adults, the need for revaccination and the optimal intervals for revaccination to maintain population protection. Current pneumococcal vaccination strategies should be continuously monitored and assessed for their effectiveness and utility against the changing epidemiology of Sp-related diseases, especially among adults and the elderly.
